# Optimization of an automated FI-FT-IR procedure for the determination of *o*-xylene, toluene and ethyl benzene in *n*-hexane

**DOI:** 10.1155/S1463924699000139

**Published:** 1999

**Authors:** Ian Wells, Paul J. Worsfold

**Affiliations:** Department of Environmental Sciences Plymouth Environmental Research Centre University of Plymouth, Drake Circus Devon Plymouth PL4 8AA UK

## Abstract

The development and optimization of an automated flow injection (FI) manifold coupled with a Fourier transform infrared (FT-IR) detector for the determination of toluene, ethyl benzene and *o*-xylene in an *n*-hexane matrix is described. FT-IR parameters optimized were resolution and number of co-added scans; FI parameters optimized were type of pump tubing, carrier flow rate and sample volume. ATR and transmission flow cells were compared for the determination of *o*-xylene, the ATR cell was easier to use and gave better figures of merit, except for sensitivity, for which the transmission cell was twice as good. Multivariate calibration routines were applied to the FI-FT-IR data and the PLS1 algorithm gave relative root mean standard errors of crossvalidation (RRMSECVs) < 7% for all three analytes using mean-centred data and the first derivative for *o*-xylene.


Journal of Automated Methods & Management in Chemistry, Vol. 21, No. 4 (July–August 1999) pp. 113–119

### Optimization of an automated FI-FT-IR procedure for the determination of *o*-xylene, toluene and ethyl benzene in *n*-hexane

**Ian Wells and Paul J. Worsfold**[1]

*Department of Environmental Sciences, Plymouth Environmental Research Centre, University of Plymouth, Drake Circus, Plymouth, Devon PL4 8AA, UK.* [1]*e-mail: pworsfold@plymouth.ac.uk*

*The development and optimization of an automated flow injection (FI) manifold coupled with a Fourier transform infrared (FT-IR) detector for the determination of toluene, ethyl benzene and o-xylene in an n-hexane matrix is described. FT-IR parameters optimized were resolution and number of co-added scans; FI parameters optimized were type of pump tubing, carrier flow rate and sample volume. ATR and transmission flow cells were compared for the determination of o-xylene, the ATR cell was easier to use and gave better figures of merit, except for sensitivity, for which the transmission cell was twice as good. Multivariate calibration routines were applied to the FI-FT-IR data and the PLS1 algorithm gave relative root mean standard errors of cross-validation (RRMSECVs) < 7% for all three analytes using mean-centred data and the first derivative for o-xylene.*

### Introduction

Flow injection (FI) is a potentially useful front-end technique for FT-IR in terms of automated sample induction and on-line physical/chemical treatment [1]. Since the earliest reported combination of FI with FT-IR in 1991 [2–4] there have been a limited number of publications on the subject, principally by de la Guardia and co-workers [5–15] and Kellner and co-workers [16–21].

During this period, there have also been several accounts of the application of multivariate calibration (MVC) routines to FT-IR data (as distinct from NIR data) for quantification. Examples of partial least squares (PLS) regression techniques applied to FT-IR data include: monitoring the epoxidation of indene to indene oxide [22], and the hydrolysis of penicillin V to 6-animopenicillanic acid [23]; the determination of methanol and methyl tertiary butyl ether in gasolines [24]; chlorinated hydrocarbons in water [25], and glucose, maltose and fructose in sugar syrups [26]; and comparison of PLS with multiple linear regression (MLS) and principal components regression (PCR) for quantifying latex in paper coatings [27].

There have, however, been very few publications that have combined the sample handling power of FI with the data treatment capabilities of MVC for organic analysis using FT-IR [1, 2]. The combination has excellent potential for the characterization/quantification of organic process streams but does require an integrated and automated system for reliable data acquisition. This paper presents a structured approach to optimizing an automated FI-FT-IR system, with data treatment using MVC techniques. The determination of benzene, toluene and *o*-xylene in hexane has been used as an industrially relevant model system to illustrate the approach.

### Experimental

### *Reagents*

Solutions of *o*-xylene (HPLC grade: Sigma-Aldrich, Gillingham, UK), toluene (AnalaR grade: Merck, Darmstadt, Germany) and ethyl benzene (GPR grade: Sigma, Poole, UK) were prepared volumetrically in *n*-hexane (HPLC grade: Rathburn, Walkerburn, Scotland). Univariate calibration standards for *o*-xylene, toluene and ethyl benzene (0–20% v/v) were prepared by addition of the appropriate volume of analyte into a volumetric flask (Grade A) and making up to the mark with *n*-hexane. Standards were prepared in a cool, unheated room to minimize volatilization. For multivariate data analysis, a full factorial design was used where the three analytes were represented at three concentrations (0, 10 and 20% v/v), producing 27 calibration standards.

### *Instrumentation*

The FI manifold consisted of two peristaltic pumps (Ismatec Mini-S 820 for sample flow and Gilson Minipuls for carrier flow) and a low-pressure injection valve (Rheodyne 5020) operated manually or automatically (Universal valve switching module). Pump tubing of 1.3 mm i.d. (Ismatec Viton) was used for sample and carrier flow. PTFE tubing of 0.8 mm i.d. was used for all flow lines. A Fourier transform mid-IR spectrometer (Bruker IFS-66) fitted with a closed cycle water-cooled globar source, Ge/KBr beamsplitter (6500–400 $cm^{-1}$), Michelson interferometer with air bearing scanner (maximum resolution 0.25 $cm^{-1}$) and a mercury cadmium telluride detector (7000–420 $cm^{-1}$) were used throughout. The instrument atmosphere was supplied by an air drier (Balston 75-52) at a flow rate of 3.4 $l\,min^{-1}$. Carbon dioxide was reported as being less than 2 ppm. One of two liquid cells was used to present the sample to the spectrometer: a Squarecol microflow through ZnSe ATR 330 µl cell (Graseby Specac); or a microflow through transmittance cell (Graseby Specac) equipped with KBr windows and 0.05 mm Pb spacer.

The ATR cell was used unless otherwise stated. Spectra were acquired using the instrument operating software (Bruker OPUS/IR-2 extended analytical package) with additional application packages (Bruker OPUS/CHROM chromatographic coupling package and OPUS/3D data processing package) required for monitoring samples introduced via the FI manifold.

Post-experimental data processing was performed using OPUS/IR-2, spreadsheets (Microsoft Excel v5.0, v7.0 and MicroCal Origin v3.78) and a multivariate data analysis package (Camo Unscrambler v6.1). Automation routines were written within OPUS/IR-2, and external apparatus control was performed via transistor–transistor logic (TTL) signals sent from the spectrometer via a custom-built interface. For automated data collection [1], a macro program was initiated and samples introduced to the FI manifold automatically. The macro was used to control external apparatus by switching one of four TTL lines to 0 V or +5 V according to the device required. The carrier pump, sample pump and injection valve were switched on and off via an in-house built TTL to mains converter. A direct TTL signal was used to actuate the valve between the load and inject positions during analysis.

### *Procedures*

For optimization of the FI-FT-IR manifold, the basic FT-IR parameters used were single-sided fast return acquisition of the interferogram with Blackman Harris 3 Term apodization and Mertz phase correction. The zero filling factor was set to 2 with a phase resolution of 32, and the aperture was opened to the maximum allowed without saturating the detector. Prior to acquiring a sample spectrum, a background spectrum of the *n*-hexane carrier was acquired. Two measurement modes were employed: (i) continuous—where the throughput of the FI manifold was constantly monitored; and (ii) automated—where discrete spectra were acquired only when the injected sample was present in the flow cell.

For continuous measurement, OPUS/CHROM software was used to continuously acquire spectra and display them as a three-dimensional output (absorbance and wavenumber as a function of time) for the duration of the experiment. Quantitative data were acquired by integrating the absorbance profile of a C–H bending mode of each analyte (*o*-xylene = 741 $cm^{-1}$, toluene = 728 $cm^{-1}$, ethyl benzene = 698 $cm^{-1}$), and plotting the integration value as a function of time to generate a FIagram. Continuous measurement was used to estimate the optimized parameters for automated measurements.

For automated measurements, a macro was written to coordinate sample injection with spectral acquisition. Upon initiation of the macro, the FI manifold was flushed for 20 s and a background spectrum of 50 co-added scans acquired, after which the sample was injected into the carrier stream. After a delay of 18 s, a sample spectrum of 50 co-added scans was acquired and integrated. This enabled a spectrum of optimum signal-to-noise ratio to be acquired whilst the central portion of the injected sample passed through the flow cell. To determine the time delay required before sample injection, five repeat injections of a 5% v/v *o*-xylene standard were performed using the procedure for continuous measurement. The delay was calculated as (time from injection to maximum FIagram response) – (half of total measurement time).

Each injection and measurement was replicated three times, background corrected against a 100% v/v *n*-hexane blank, and averaged to produce a mean spectrum. No smoothing was applied to raw spectral data and all samples were measured in a randomized order to reduce the risk of systematic error.

### Results and discussion

### *Choice of pump tubing*

There are a number of materials available for use as pump tubes in peristaltic pumps. The choice of material is primarily dictated by operating life and chemical resistance to the carrier stream. Small samples of five types of commercially available pump tubing (Ismaprene®, Silicone®, Viton®, Tygon® and Tygon Yellow®) were immersed in 10 ml of *n*-hexane in aluminium-capped bottles for 72 h and tested for changes in internal/external diameter and hardness (using the International Rubber Hardness Degrees scale (IRHD)) (table 1). Silicone was found to expand and soften in *n*-hexane to give an IRHD value below the limit of detection of the instrument. Ismaprene® and Tygon® contracted, with the former showing a 25% reduction in hardness and the latter hardening beyond the upper measurable limit. These three pump tubing materials were therefore not suitable for pumping *n*-hexane. The remaining materials, Tygon Yellow® and Viton® were further assessed for suitability by monitoring their flow rates over 70 h of continuous pumping using an Ismatec Mini-S 820 peristaltic pump. The results showed that long-term deployment of Tygon Yellow® was not feasible as the pump tube walls collapsed within 5 h. Viton® pumped *n*-hexane at a constant rate for 70 h and was

*Table 1. Changes in i.d., e.d. and IRHD of various pump tubing materials before and after immersion in n-hexane for 72 hs.*

| *Material* | *ID before (mm)* | *ID after(mm)* | *ED before (mm)* | *ED after (mm)* | *IRHD before* | *IRHD after* |
|---|---|---|---|---|---|---|
| Ismaprene | 1.03 | 1.00 | 2.72 | 2.74 | 56 | 42 |
| Silicone | 0.98 | 0.96 | 2.94 | 3.06 | 40 | — |
| Viton | 1.09 | 1.08 | 2.97 | 2.98 | 44 | 53 |
| Tygon | 1.01 | 0.95 | 2.82 | 2.45 | 53 | — |
| Tygon Yellow | 1.00 | 0.98 | 2.74 | 2.76 | 40 | 41 |

therefore selected as the ideal pump tubing for transferring *n*-hexane matrices in all subsequent experiments.

### *Effect of instrument resolution*

The spectra of 5% v/v solutions of the individual components recorded at a resolution of 2 cm$^{-1}$ showed (figure 1) the C–H deformation bands for ethyl benzene (698 cm$^{-1}$), *o*-xylene (741 cm$^{-1}$) and toluene (728 cm$^{-1}$). The spectra of a 5% v/v mixture recorded at resolutions of 2, 4, 8, 16 and 32 cm$^{-1}$ are shown in figure 2. At a resolution of 2 cm$^{-1}$, the three deformation bands were well resolved and there was some low-level spectral detail in the window 1150–1000 cm$^{-1}$. Reducing the resolution to 4 cm$^{-1}$ decreased the separation between the major *o*-xylene (741 cm$^{-1}$) and toluene (728 cm$^{-1}$) bands, but the combined ethyl benzene and toluene band (696 cm$^{-1}$) was still clearly separated. Reducing the resolution further to 8, 16 and 32 cm$^{-1}$ degraded the quality of spectral resolution to the point where, at 32 cm$^{-1}$, all three deformation bands were indistinguishable. A resolution of 4 cm$^{-1}$ was subjectively estimated to provide adequate spectral information for the purposes of calibration.

In terms of measurement time, a resolution of 4 cm$^{-1}$ was much faster (8.1 s) than a resolution of 2 cm$^{-1}$ (14.2 s) in acquiring a spectrum of eight co-added scans at a modulation frequency of 10.0 kHz. Increasing the modulation frequency to 160.0 kHz reduced the measurement time considerably to 0.6 and 0.9 s for 4 cm$^{-1}$ and 2 cm$^{-1}$, respectively. A resolution of 4 cm$^{-1}$ and a modulation frequency of 160 kHz were therefore used in all subsequent experiments to maximize the number of co-added scans acquired during a measurement event.

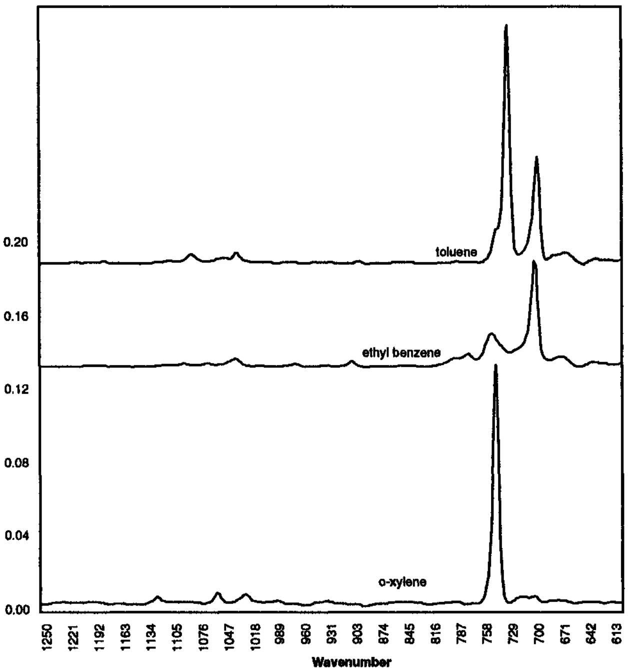

*Figure 1. Spectra of 5% v/v samples of toluene, o-xylene and ethyl benzene in n-hexane. Spectra are offset for clarity.*

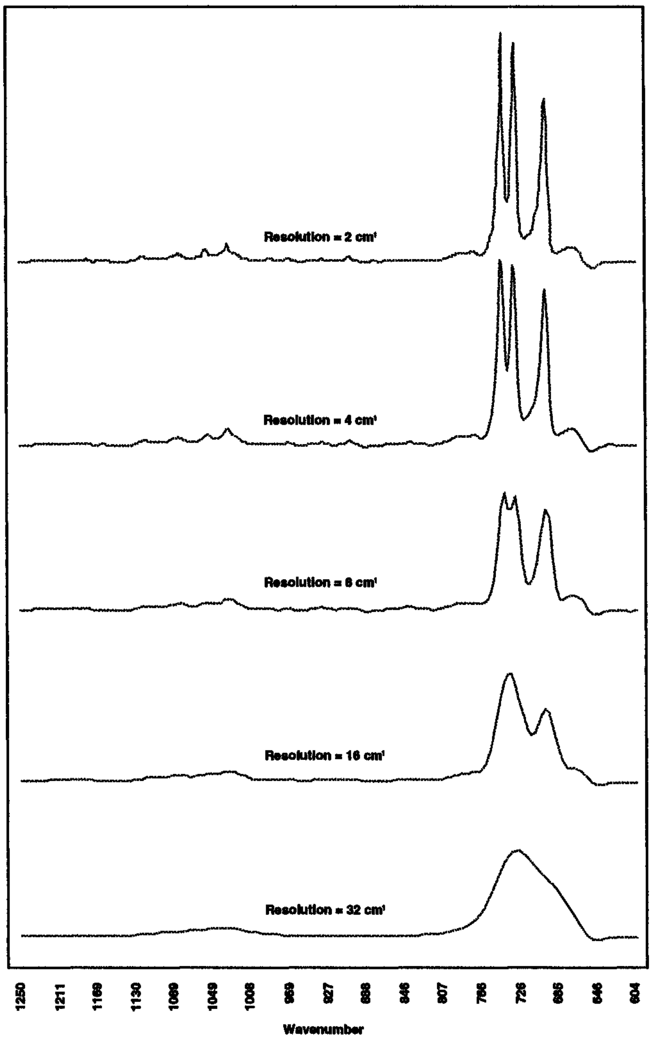

*Figure 2. Effect of instrument resolution on the spectrum of a 5% v/v mixture of toluene, ethyl benzene and o-xylene. Spectra are offset for clarity.*

### *Spectral noise as a function of the number of co-added scans and instrument resolution*

To assess spectral noise as a function of the number of co-added scans, three replicate spectra of 5% v/v mixture solutions in *n*-hexane were acquired by co-adding 1, 10, 50 100, 250, 500 and 1000 scans at a resolution of 4 cm$^{-1}$. Maximum peak-to-peak noise was determined in the range 2100–2000 cm$^{-1}$, where there was no absorbance from the analytes, leaving a signal due only to fluctuations in the instrument and flow rate of the carrier stream. As expected, noise and reproducibility (shown as ± one standard deviation) decreased with increasing number of co-added scans, as shown in figure 3. No significant reduction in noise was observed with increasing the number of co-added scans above 10. Fifty co-added scans gave an RSD of 3.2% and this was selected as the optimum condition for discrete spectra.

To study the effect of instrument resolution on spectral noise, three replicate 50 scan spectra of 5:5:5% v/v mixture solutions in *n*-hexane were acquired at resolutions of 2, 4, 8, 16 and 32 cm$^{-1}$. Maximum peak-to-peak noise (calculated as above) and reproducibility decreased

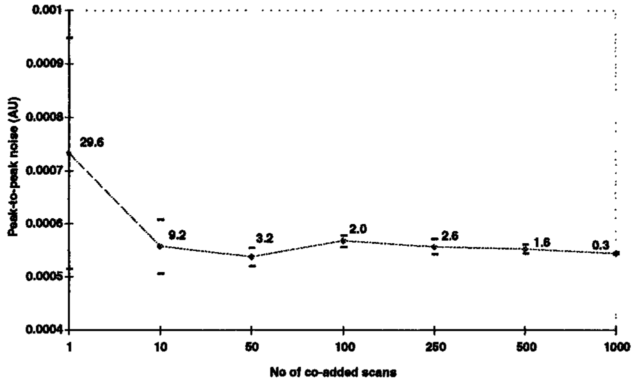

*Figure 3. Peak-to-peak noise as a function of the number of co-added scans. Figures indicate RSD (%) for noise over three replicate spectra.*

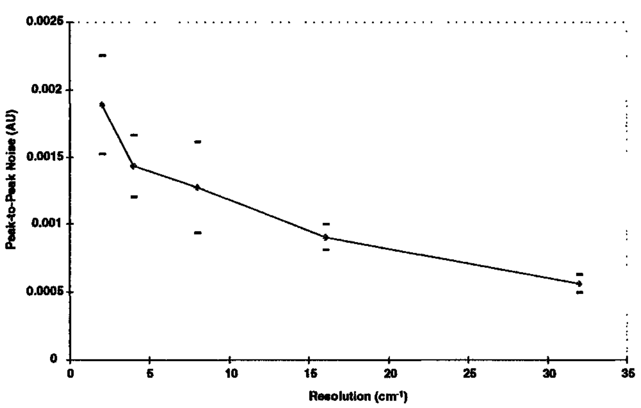

*Figure 4. Peak-to-peak noise as a function of instrument resolution.*

with coarser resolution (figure 4). A resolution of 4 $cm^{-1}$ was therefore selected as the optimum condition.

### *Effect of FI parameters*

In FI with spectrophotometric detection of chemically derivatized analytes decreasing the flow rate decreases sample dispersion and increases residence time in the FI manifold, which improves sensitivity for slow reactions. With FT-IR detection of organic species, however, derivatization is not normally required. In addition, the residence time of the sample in the beam path directly affects the number of sample scans that can be acquired, which in turn affects the signal-to-noise ratio. Flow rate should therefore be optimized so that the sample is resident in the flow cell long enough to achieve the required signal-to-noise ratio.

Five replicate 20 µl injections of a 5% v/v *o*-xylene standard were manually injected into an *n*-hexane carrier stream at flow rates of 0.5, 1.0, 1.5 and 2.0 ml $min^{-1}$. The response was monitored continuously based on measurement of the peak height of the *o*-xylene band at 741 $cm^{-1}$. Spectra of eight co-added scans were continuously acquired, and all sample spectra were ratioed against a background spectrum of 100 co-added scans of 100% v/v *n*-hexane. The reproducibilities ($n = 5$) were in the range 0.4–1.7% and there was no significant difference in signal response with varying carrier flow rate for a 20 µl sample volume. This is contrary to expectation for transmission cells [28, 29] and was due to the small volume of the injected sample (20 µl) relative to that of the ATR flow cell (330 µl). Hence, a carrier flow rate of 2.0 ml $min^{-1}$ was used to maximize throughput.

Five replicates of 25, 100, 150, 200 and 300 µl volumes of a 5% v/v *o*-xylene standard were manually injected into an *n*-hexane carrier stream pumped at a flow rate of 2.0 ml $min^{-1}$. The response increased linearly with sample loop volume, but the larger volumes took longer to return to the baseline. The increase in response was also due to the large volume of the ATR flow cell compared with the range of sample volumes studied, i.e. larger sample volumes covered a larger area of the ATR element. The optimum sample volume selected was 220 µl which was a compromise between sensitivity and fast return to baseline.

### *FI-FT-IR calibration for toluene, ethyl benzene and o-xylene*

With the data acquisition and FI parameters sequentially optimized, the analytical performance of the FI-FT-IR system was assessed by examining the calibration data for the analytes in *n*-hexane. Five replicate injections of 200 µl of a range of toluene (0.04–20% v/v), ethyl benzene (0.04–20% v/v) and *o*-xylene standards (0.05–20% v/v) were manually injected into an *n*-hexane carrier flow stream at a flow rate of 2.0 ml $min^{-1}$. The response was measured continuously as described above. The linear calibration parameters are given in table 2 with limits of detection (LOD) defined as three times the standard deviation of the lowest standard because blank injections could not be detected on the carrier baseline. The large relative standard errors (13–18%) observed at low concentrations could be minimized by co-adding a higher number of scans to improve the signal-to-noise ratio. However, increasing the number of scans in time-resolved measurements limited the temporal resolution of the FIagram response. The preferred approach to solving this problem is therefore to automate the FI-FT-IR manifold. A further advantage of automation is that computer memory consumption is significantly reduced; a time-resolved measurement takes up to 5 Mb of hard disk space, whereas discrete single spectrum measurements require only 10 kb.

### *Automated FI-FT-IR calibration for toluene, ethyl benzene and o-xylene*

A macro was written to control external apparatus via a 50 pin serial D connector and a 5 V TTL signal. Five replicates of 200 µl of a range of toluene, ethyl benzene and *o*-xylene standards (0–20% v/v) were injected into an *n*-hexane carrier stream pumped at 2.0 ml $min^{-1}$. All sample spectra were ratioed against a background spectrum of 500 co-added scans of the carrier stream, 100% v/v *n*-hexane. A higher degree of repeatability (< 7% error) was attained for standards of concentrations greater than 0.05% v/v with the automated manifold,

*Table 2. Parameters for the calibration of toluene, ethyl benzene and o-xylene by FI-FT-IR.*

| *Analyte* | *FI manifold* | *Analytical wavenumber ($cm^{-1}$)* | *Baseline points for integration ($cm^{-1}$)* | *Sensitivity (AU/% v/v)* | *Intercept (AU)* | $r^2$ | *LOD (% v/v)* |
|---|---|---|---|---|---|---|---|
| Toluene | Manual | 728 | 757–715 | 0.0377 | 0.0125 | 0.994 | 0.030 |
| | automated | | | 0.0291 | 0.00416 | 0.999 | 0.014 |
| Ethyl | Manual | 698 | 715–680 | 0.0162 | 0.00342 | 0.999 | 0.020 |
| benzene | automated | | | 0.0147 | 0.00374 | 0.998 | 0.010 |
| *o*-Xylene | Manual | 741 | 760–727 | 0.0372 | −0.00089 | 0.997 | 0.020 |
| | automated | | | 0.0352 | 0.000775 | 0.999 | 0.004 |

demonstrating that repeatability improved compared to the manual approach. The calibration results are shown in table 2, and the limit of detection of 0.004% v/v for *o*-xylene in *n*-hexane compares favourably with previously reported values of 0.03% v/v [14] and 0.01% v/v [15] using a manual FI-FT-IR manifold equipped with a transmission flow cell.

*Comparison of ATR and transmittance flow cells*

The two alternatives for presenting samples to an IR spectrometer from an FI manifold are ATR and transmittance flow cells. Previous results [2] have shown that whilst transmittance flow cells have a higher throughput of source energy than ATR cells, they are difficult to assemble and maintain. ATR flow cells are more mechanically robust and optically stable due to their fixed path lengths. A micro flow-through transmittance cell was incorporated into the FI manifold in place of the ATR cell, and the manifold optimized for flow rate and sample loop volume. Five replicates of 20 μl of a 5% v/v *o*-xylene standard were manually injected into an *n*-hexane carrier stream at flow rates of 0.5, 1.0, 1.5 and 2.0 ml $min^{-1}$. In addition, five replicates of 25, 100, 150, 200 and 300 μl of a 5% v/v *o*-xylene standard were manually injected into an *n*-hexane carrier stream at a flow rate of 0.5 ml $min^{-1}$. The response in both experiments was measured continuously. Absorbance was inversely related to flow rate, with a maximum mean observed signal of 0.31 AU at 0.5 ml $min^{-1}$. The repeatability error of sample response ($n = 5$) was less than 0.7% in all instances. The observation of the maximum response occurring at the minimum carrier flow rate was expected as sample dilution due to axial dispersion in the FI manifold was minimized. This is in contrast to the flow through ATR cell where flow rate had no significant effect on sample response. A carrier flow rate of 0.5 ml $min^{-1}$ was selected for all subsequent experiments where the transmittance flow cell was used. The effect of sample loop volume in the FI manifold on sample response followed a similar trend as observed for the flow through ATR cell, with the transmittance flow cell exhibiting a greater sensitivity. Repeatability for a 5% v/v *o*-xylene standard ($n = 5$) was less than 0.6% for all sample loop volumes investigated. The optimum sample loop volume was 200 μl, as this gave a high response with an acceptably fast return to the baseline. A regression coefficient ($r^2$) of 0.9999, a sensitivity of 0.069 AU (% v/v *o*-xylene)$^{-1}$ and an intercept of 0.0032 AU were obtained over the range 0–20% v/v *o*-xylene.

The figures of merit for automated *o*-xylene calibration using the ATR and transmittance cells are shown in table 3 and were similar for most parameters. Overall, the ATR cell was the more user-friendly accessory, being easier to dismantle, clean and re-assemble. The transmittance cell suffered from leaks if the entrance and exit ports were not correctly aligned with the flow-through KBr chamber (alignment was best achieved with the insertion of a thin wire guide through the ports prior to tightening). Optical alignment is crucial with most ATR accessories for maximizing sensitivity. Adjustments have to be made in all three dimensions and fine-tuning performed until no further gains can be made in signal throughput, which is time consuming and labour intensive. In contrast, the transmittance cell requires the minimum of alignment. ATR cells are highly reproducible once aligned due to their fixed path length, but reproducibility in transmittance cells is difficult to achieve as the path length changes with the tension applied to the fastening nuts of the demountable cell. The ATR cell used in this investigation suffered from poor flushing efficiency due to the orientation of the ZnSe crystal and the entrance and exit ports. No such effect was observed in the simpler transmittance cell. The choice of flow cell is ultimately dependent on the sample matrix, e.g. acid media cannot be used with ZnSe ATR elements due to the production of toxic gaseous $H_2Se$. For analytical purposes the ATR cell is preferred due to better reproducibility and ease of use.

*Table 3. Comparison of performance characteristics of an ATR and a transmittance flow cell used in an FI-FT-IR manifold for the determination of o-xylene in n-hexane.*

| *Parameter* | *ATR cell* | *Transmittance cell* |
|---|---|---|
| Sensitivity [AU(% v/v *o*-xylene)$^{-1}$] | 0.035 | 0.069 |
| LOD (% v/v *o*-xylene) | 0.0043 | 0.0026 |
| Intercept (AU) | 0.00078 | 0.0032 |
| Linear range (% v/v *o*-xylene) | 0–20 | 0–20 |
| RSD (0.05% v/v *o*-xylene) | 5.16 | 0.40 |
| RSD (5% v/v *o*-xylene) | 0.86 | 0.41 |
| RSD (20% v/v *o*-xylene) | 0.70 | 0.23 |
| Flow rate (ml $min^{-1}$) | 2.01 | 0.51 |
| Sample volume (μl) | 200 | 200 |
| Throughput ($h^{-1}$) | 60 | 36 |
| $r^2$ calibration coefficient (0–20% v/v *o*-xylene) | 0.9998 | 0.9999 |

*Table 4. Model development for the multivariate calibration of a full factorial designed mixture of toluene, ethyl benzene and o-xylene in n-hexane using the PLS1 algorithm.*

| *Preprocessing* | *Wavenumber range ($cm^{-1}$)* | *RRMSECV toluene (%)* | *RRMSECV ethyl benzene (%)* | *RRMSECV o-xylene (%)* |
|---|---|---|---|---|
| None | 2000–600 | 8.7 (3) | 4.7 (3) | 6.9 (3) |
| MC | 2000–600 | 7.7 (3) | 4.4 (3) | 6.2 (3) |
| MC, AS | 2000–600 | 10.5 (2) | 5.5 (3) | 4.9 (3) |
| MC, Norm | 2000–600 | 55.0 (4) | 17.8 (5) | 40.0 (1) |
| MC, Drv1 | 1997–600 | 9.8 (2) | 6.0 (2) | 4.3 (2) |
| MC, Drv2 | 1995–600 | 10.3 (1) | 5.8 (2) | 4.0 (2) |

Number of PCs used in calibration shown in parentheses. (MC—mean centring; AS—autoscaling; Norm—mean normalization; Drv1—first derivative; Drv2—second derivative.)

*Multivariate calibration for toluene, ethyl benzene and o-xylene using a full factorial experimental design*

PLS1 calibration was used to model a full factorial ($3^3$) experimental design of toluene, ethyl benzene and *o*-xylene at concentrations of 0, 10 and 20% v/v. Three replicate injections of 200 µl of the standards were injected into an *n*-hexane carrier flow stream at a flow rate of 2.0 ml min$^{-1}$, and spectra acquired with the automated and optimized FI manifold equipped with the ATR flow cell. All sample spectra were ratioed against a background spectrum of 500 co-added scans of the carrier stream, 100% v/v *n*-hexane. Mean spectra were calculated for each standard and imported into Unscrambler v6.1 for multivariate data analysis.

Exploratory data analysis by principal components analysis (PCA) revealed a well-defined structure to the data. The scores of the first three principal components (PCs) showed samples 1–9, 10–18 and 19–27 clearly grouped according to the concentration of the analytes and the co-varying solvent in the experimental design. Loadings of the first PC approximated, as expected, to a spectrum of a mixture of toluene, ethyl benzene and *o*-xylene in *n*-hexane. No outliers were observed and therefore multivariate calibration was performed as the next step of the analysis.

The results of applying the PLS1 algorithm to the factorially designed calibration set are shown in table 4. Mean centring was found to be beneficial. Optimum relative root mean standard error of cross-validation (RRMSECV) [1] errors of 7.7% for toluene and 4.4% for ethyl benzene were observed for three PC models based on mean centring only. The best *o*-xylene model required second derivatization and only two PCs to produce an RRMSECV of 4.0%. The results show that rapid FT-IR data acquisition of liquid samples is possible through the use of FI, and that the results can be used for quantitative prediction via univariate and multivariate calibration.

### Conclusions

The optimized and automated FI manifold provided a rapid and reproducible means of liquid sample introduction to FT-IR. The manifold used for the determination of toluene, ethyl benzene and *o*-xylene in *n*-hexane was optimized for the following parameters: pump tubing (Viton), resolution (4 cm$^{-1}$), number of co-added scans (50), modulation frequency (160 kHz), flow rate (2 ml min$^{-1}$ for the ATR cell, 0.5 ml min$^{-1}$ for the transmission cell) and sample loop size (200 µl). Each of the analytes was univariately calibrated in binary mixtures (0–20% v/v) with *n*-hexane. Limits of detection were 0.014% v/v for toluene, 0.010% v/v for ethyl benzene and 0.004% v/v for *o*-xylene, and the error of repeatability was $<7\%$ for concentrations greater than 0.05% v/v. The analytical performance of an ATR and transmission flow cells were compared using an *o*-xylene calibration, and the ATR cell was more user-friendly and analytically superior, except for sensitivity, which was approximately half that of the transmission cell.

The use of PLS1 for the multivariate calibration of mixture spectra of toluene, ethyl benzene and *o*-xylene in *n*-hexane acquired by automated FI-FT-IR resulted in RRMSECVs of $<8\%$. Derivatization was required to enhance the resolution of the acquired spectra, at the cost of much poorer signal-to-noise ratio, for the prediction of *o*-xylene.

### Acknowledgments

The authors gratefully acknowledge financial support from EPSRC and Zeneca via the CASE scheme, and technical support from Graseby Specac.

### References

1. Kellner, R., Lendl, B., Wells, I. and Worsfold, P. J., *Applied Spectroscopy*, **51** (1997), 227.
2. Guzman, M., Ruzicka, J., Christian, G. D. and Shelley, P., *Vibrational Spectroscopy*, **2** (1991), 1.
3. McKittrick, P. T., Danielson, N. D. and Katon, J. E., *Microchemical Journal*, 44 (1991), 105.
4. Huber, S. A. and Frimmel, F. H., *Analytical Chemisty*, **63** (1991), 2122.
5. SanchezDasi, M. J., Garrigues, S., Cervera, M. L. and de la Guardia, M., *Analytica Chimica Acta*, **361** (1998), 253.
6. Bouhsain, Z., Garrigues, S. and de la Guardia, M., *Analyst*, **122** (1997), 441.

7. Daghbouche, Y., Garrigues, S., Vidal, M. T. and de la Guardia, M., *Analytical Chemistry*, **69** (1997), 1086.

8. Daghbouche, Y., Garrigues, S. and de la Guardia, M., *Analyst*, **121** (1996), 1031.

9. Bouhsain, Z., Garrigues, S. and de la Guardia, M., *Analyst*, **121** (1996), 635.

10. Gallignani, M., Garrigues, S., de la Guardia, M., Burguera, J. L. and Burguera, M., *Talanta*, **41** (1994), 739.

11. Garrigues, S., Gallignani, M. and de la Guardia, M., *Talanta*, **40** (1993), 1799.

12. de la Guardia, M., Gallignani, M. and Garrigues, S., *Analytica Chimica Acta*, **282** (1993), 543.

13. Gallignani, M., Garrigues, S. and de la Guardia, M., *Analytica Chimica Acta*, **274** (1993), 267.

14. Garrigues, S., Gallignani, M. and de la Guardia, M., *Analyst*, **117** (1992), 1849.

15. de la Guardia, M., Garrigues, S., Gallignani, M., Burguera, J. L. and Burguera, M., *Analytica Chimica Acta*, **261** (1992), 53.

16. Schindler, R., Lendl, B. and Kellner, R., *Analyst*, **122** (1997), 531.

17. Vonach, R., Lendl, B. and Kellner, R., *Analyst*, **122** (1997), 525.

18. Kellner, R. and Lendl, B., *Analytical Methods and Instrumentation*, **2** (1995), 52.

19. Lendl, B. and Kellner, R., *Mikrochimica Acta*, **119** (1995), 73.

20. Rosenberg, E. and Kellner, R., *Fresenius Journal of Analytical Chemistry*, **348** (1994), 530.

21. Rosenberg, E. and Kellner, R., *Vibrational Spectroscopy*, **5** (1993), 33.

22. Ge, Z., Thompson, R., Cooper, S., Ellison, D. and Tway, P., *Process Control and Quality*, **7** (1995), 3.

23. Guzman, M., de Bang, M., Ruzicka, J. and Christian, G. D., *Process Control and Quality*, **2** (1992), 113.

24. Garcia, F. X., de Lima, L. and Medina, J. C., *Applied Spectroscopy*, **47** (1993),1036.

25. Conzen, J. P., Burck, J. and Ache, J. P., *Fresenius Journal of Analytical Chem*istry, **348** (1994), 501.

26. de Lene Mirouze, F., Boulou, J. C., Dupuy, N., Meurens, M., Huvenne, J. P. and Legrand, P., *Applied Spectroscopy*, **47** (1993), 1187.

27. Dupuy, N., Duponchel, L., Amram, B., Huvenne, J. P. and Legrand, P., *Journal of Chemometrics*, **8** (1994), 333.

28. Ruzicka, J. and Hansen, E. H., *Flow Injection Analysis* (New York: Wiley) (1981).

29. Valcarcel, M. and Luque de Castro, M. D., *Flow Injection Analysis, Principles and Applications* (Chichester: Ellis Horwood) (1987).

